# Tofacitinib Downregulates TNF and Poly(I:C)-Dependent MHC-II Expression in the Colonic Epithelium

**DOI:** 10.3389/fimmu.2022.882277

**Published:** 2022-05-17

**Authors:** Shreya Gopalakrishnan, Marianne Doré Hansen, Helene Kolstad Skovdahl, Ingrid Aass Roseth, Atle van Beelen Granlund, Ann Elisabet Østvik, Ingunn Bakke, Arne Kristian Sandvik, Torunn Bruland

**Affiliations:** ^1^ Department of Clinical and Molecular Medicine (IKOM), NTNU - Norwegian University of Science and Technology, Trondheim, Norway; ^2^ Department of Gastroenterology and Hepatology, Clinic of Medicine, St. Olav’s University Hospital, Trondheim, Norway; ^3^ Clinic of Laboratory Medicine, St. Olav’s University Hospital, Trondheim, Norway; ^4^ Centre of Molecular Inflammation Research (CEMIR), NTNU - Norwegian University of Science and Technology, Trondheim, Norway

**Keywords:** intestinal epithelium, organoids, tumor necrosis factor (TNF), polyinosinic:polycytidylic acid Poly(I:C), antigen presentation, major histocompatibility class II, tofacitinib, ulcerative colitis

## Abstract

Major Histocompatibility Complex (MHC)-I and -II genes are upregulated in intestinal epithelial cells (IECs) during active inflammatory bowel diseases (IBD), but little is known about how IBD-relevant pro-inflammatory signals and IBD drugs can regulate their expression. We have previously shown that the synthetic analog of double-stranded RNA (dsRNA) Polyinosinic:polycytidylic acid (Poly(I:C)), induces interferon stimulated genes (ISGs) in colon organoids (colonoids). These ISGs may be involved in the induction of antigen presentation. In the present study, we applied colonoids derived from non-IBD controls and ulcerative colitis patients to identify induction and effects of IBD-drugs on antigen presentation in IECs in the context of Tumor Necrosis Factor (TNF)-driven inflammation. By RNA sequencing, we show that a combination of TNF and Poly(I:C) strongly induced antigen-presentation gene signatures in colonoids, including expression of MHC-II genes. MHC-I and -II protein expression was confirmed by immunoblotting and immunofluorescence. TNF+Poly(I:C)-dependent upregulation of MHC-II expression was associated with increased expression of Janus Kinases *JAK1/2* as well as increased activation of transcription factor Signal transducer and activator of transcription 1 (*STAT1*). Accordingly, pre-treatment of colonoids with IBD-approved pan-Janus Kinase (JAK) inhibitor Tofacitinib led to the downregulation of TNF+Poly(I:C)-dependent MHC-II expression associated with the abrogation of STAT1 activation. Pre-treatment with corticosteroid Budesonide, commonly used in IBD, did not alter MHC-II expression. Collectively, our results identify a regulatory role for IBD-relevant pro-inflammatory signals on MHC-II expression that is influenced by Tofacitinib.

## Introduction

Inflammatory bowel diseases (IBD) encompassing Crohn’s disease (CD) and ulcerative colitis (UC) are associated with chronic relapsing intestinal inflammation. The pathobiology of IBD is incompletely understood but involves a disturbed crosstalk between intestinal epithelium, microbiota and host immune system in a genetically susceptible host (1, 2). Much of the focus in the past has been drawn to investigating the involvement of immune cells in IBD pathobiology. More recently, the intestinal epithelial cells (IECs) are emerging as crucial immune modulators and identified as an essential target in IBD treatment (3).

As immune modulators, IECs are non-professional antigen-presenting cells that present self and antigenic peptides to the host immune cells *via* the expression of Major Histocompatibility Class (MHC) I and II complexes. Three different isoforms of MHC-II exist in humans: HLA-DP, HLA-DR and HLA-DQ, encoded by α and β chains and their expression is highly controlled by MHC-II transactivator CIITA (4, 5). Once synthesized, the MHC-II molecules assemble in the endoplasmic reticulum upon association with invariant chain CD74 (6). This complex further translocates to the acidic endosomal compartment where the invariant chain is trimmed to class II invariant chain-associated peptide (CLIP) with the help of lysosomal protease cathepsin S (CTSS) (7, 8). Subsequently, the non-classical HLA-DM protein associates with CLIP to catalyze the exchange of CLIP with antigenic peptides in the peptide-binding groove of MHC-II that translocate to the cell surface for antigen presentation to the adaptive T cells (9–11).

MHC-II expression by IECs is critical for regulating CD4+ T cells in the lamina propria (12) and differentiation of intraepithelial lymphocytes (13, 14) that are found between the IECs. We (15) and others (16–18) have documented that MHC-II expression is upregulated in the colonic epithelium during active IBD. MHC-II expression in IECs is induced predominantly by Interferon gamma (IFNγ) from immune cells invading the mucosa during active inflammation in IBD (19–22). IFNγ signals *via* the Janus Kinases 1/2, Signal transducer and activator of transcription 1, and Interferon regulatory factor 1 (JAK1/2-STAT1-IRF1) pathway to upregulate MHC-II expression (23). Studies in murine models have revealed that IEC-specific MHC-II expression requires the presence of microbiota and microbial-driven Toll-like receptor signaling proteins TRIF and MYD88 (24, 25). These signals may further induce IFNγ protein expression from immune cells to upregulate MHC-II expression in IECs.

IBD is associated with enhanced Tumor necrosis factor (TNF) signaling and excessive epithelial cell death. It is unknown whether RNA signals derived from dead cells, such as damage-associated molecular patterns (DAMP), can impact antigen presentation in IECs. Additionally, whether viral infections in IBD can impact MHC-II expression is uninvestigated, particularly in the context of TNF (26). In a transcriptomic time-series screen using colonic HT-29 cells treated with TLR 1-9 ligands, NOD2 ligand, or cytokines (IL1β, IL10, TNFα), we found that TNF and TLR3 ligand Poly(I:C) that mimics viral and DAMP signals could upregulate MHC-I genes and MHC-II associated invariant chain CD74 (27). In addition, colon organoids (colonoids) stimulated with TNF and Poly(I:C) induced signature type I Interferon (IFN) Stimulated Genes (ISGs) (28). These observations suggest that pro-inflammatory signals such as from TNF or dsRNA may directly drive the expression of genes relevant to antigen presentation in colonic epithelium (15, 27).

IBD is associated with overexpression of different inflammatory cytokines, and several cytokines may be regulated *via* the JAK-STAT signaling pathway (29). Recently, small-molecule JAK inhibitors that inhibit phosphorylation and activation of STAT proteins have emerged as a promising therapy, particularly in UC patients (30–34). Currently, Tofacitinib, an oral pan-JAK inhibitor that is approved for use in UC refractory to standard treatment, has been shown to alleviate symptoms in UC patients with moderate to severe disease (31, 32, 34, 35). *Ex vivo*, Tofacitinib has been shown to intercept T-cell activation by downregulating costimulatory CD80/CD86 expression downstream to IFN-type I stimulation and LPS stimulation of dendritic cells (36). Studies in human colonoids indicates that Tofacitinib also plays a central role in the prevention of IFNγ-induced barrier defects (37). Although Tofacitinib has been shown to regulate several critical pathways of IFN signaling, the role of Tofacitinib on IEC-dependent antigen presentation has not been evaluated.

MHC-II expression in the context of IBD-associated intestinal inflammation, particularly in human colonic epithelium, remains vastly under-investigated. The roles of antigen presentation by IECs have mostly been studied in epithelial cancer cell lines which do not carry the same cellular composition as *in vivo* IECs, or in animal models (12, 14, 24, 25, 38) that do not fully reflect IBD pathobiology (39). A promising model for addressing human IBD-pathobiology and IBD-drug response in intestinal epithelium is intestinal epithelial organoids that recapitulate *in vivo* IECs-specific functional characteristics (28, 40–43). Recently, Wosen et al. (19), showed that intracellular MHC-II peptide-pathway is intact and functional in organoids from small intestine (enteroids). Here we evaluate how the expression of MHC-II genes is regulated by IBD-relevant pro-inflammatory signal TNF and Poly(I:C) in colonoids, and how they are modulated in a donor-specific manner by JAK inhibition using the IBD-approved drug Tofacitinib.

## Methods

Materials are listed in **Supplementary File SF1**, sheet 2.

### Ethical Considerations

The current study was carried out under relevant approvals by the Central Norway Regional Committee for Medical and Health Research Ethics (reference numbers 5.2007.910 and 2013/212/REKmidt). All patients included in the study provided informed written consent.

### Patient Material

Patients were admitted to the Department of Gastroenterology and Hepatology at St Olav’s University Hospital, Trondheim, Norway, for colonoscopy and clinical assessment, and were diagnosed with UC or categorized as non-IBD controls. Pinch biopsies from patients were collected in 10% buffered formalin and histopathology was evaluated as described before (44). Colonoids for functional assays were established using pinch biopsies collected from uninflamed mucosa at the hepatic flexure (28, 43). Patient characteristics are listed in **Table 1**.

**Table 1:** Colonoid donor characteristics.

**Donor (D)**	D1	D2	D3	D4	D5	D6	D7	D8	D9	D10
**Diagnosis**	Non-IBD	Non-IBD	Non-IBD	Non-IBD	Non-IBD	Non-IBD	UC	UC	UC	UC
**Age**	21	73	55	28	65	43	20	18	43	29
**Sex**	F	M	F	F	F	F	M	M	M	F
**Medication**										
**5ASA**	0	0	0	0	0	0	1	1	1	1
**Steroid**	0	0	0	0	0	0	0	1	0	1
**RNA-seq** **Figure 1**	x	x	x							
**RNA-seq** **Figure 2** and **Supplementary Figure 1**	x	X	x				x	x	x	
**Immunofluorescence** **Figure 3** and **Supplementary Figure 2**		x	x				x	x		
**Immunohistochemistry ** **Figure 3**		x	x							
**Drug treatment ** **Figure 4** and **5**				x	x	x	x	x	x	x
*In vivo* colonic biopsy for staining^*^	Hepatic flexure	NA	Hepatic flexure	Hepatic flexure	Hepatic flexure	Hepatic flexure	Cecum	Ascending colon	NA	NA
**Conclusion: MHC-II expression in colonic epithelium**	No	Yes	Yes	No	No	No	Yes	Yes	Yes	Yes

*In vivo colonic biopsies for immunostaining from the UC patiens are taken from inflamed area. All samples for colonoids are taken from Hepatic flexure. IBD, Inflammatory bowel disease; M, Male; F, Female; UC, Ulcerative colitis; 5ASA, 5-aminosalicylic acid; RNA-seq, RNA sequencing; NA, Not available.

### Colonoid Culture, Stimulation Experiments and Drug Treatment

**Figure 1:**
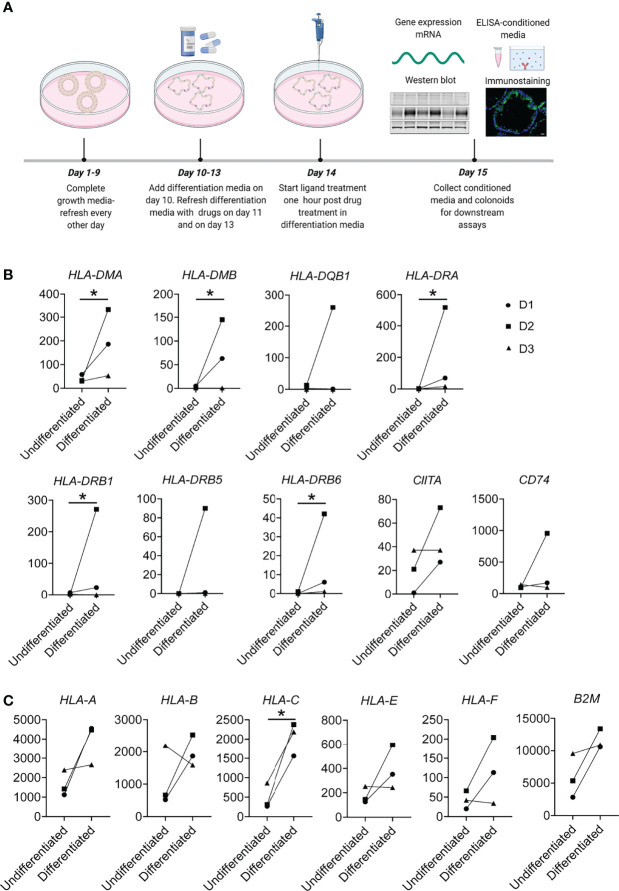
Experimental overview and MHC-II and MHC-I expression in constitutive conditions. **(A)** General graphical representation of the experiments. Colonoids were passaged and dissociated into single cells before plating for experiments. Colonoids were given complete growth media every other day where ROCK inhibitor Y-27632 was added for the first two changes, as described in the Methods section. On Day 10, colonoids received differentiation media. For experiments involving drug treatments, 50 µM Tofacitinib, 10 µM Budesonide or DMSO (vehicle controls) were added in differentiation media on day 11 and refreshed on day 13 and one hour before stimulation with ligands TNF or TNF+Poly(I:C) on day 14. On day 15, colonoids were collected for downstream experiments, and conditioned media from colonoids for analyzing protein secretion. The illustration was created with Biorender.com. **(B)** MHC-II and **(C)** MHC-I associated genes showing increased expression in differentiated colonoids compared to undifferentiated colonoids. In this experiment, colonoids from 3 non-IBD controls were passaged and plated as single cells that were cultured in growth media for 10 days and either remained in growth media for 5 additional days (undifferentiated colonoids) or differentiated with differentiation media by reducing the WNT concentration of the media. On day 15, undifferentiated and differentiated colonoids were collected and analysed for gene expression by RNAseq. The plots show paired changes in normalized mRNA reads (Y-axis) for the different donors (D1-D3, **Table 1**). *P* values were obtained by LIMMA linear models with least squares regression and empirical Bayes moderated t-statistics with Benjamini Hochberg FDR correction for multiple comparisons and * indicates *P* < 0.05.

Colonoid cultures were established and grown in 20% O_2_ and 5% CO_2_ at 37°C as described in Skovdahl et al. (43), based on protocols of Mahe et al. (40), and Jung et al. (45). Briefly, epithelial crypts isolated from biopsies were resuspended in ice-cold basement membrane matrix Matrigel GFR (Corning^®^, #734–1101, Corning, NY, USA) and 50 μL per well was added onto pre-warmed 24-well plates. Complete growth media (CGM) for the colonoids was composed of 50% Wnt-3A conditioned medium (Wnt 3A-CM, #CRL-2647, ATCC, Manassas, VA, USA), 30% Advanced DMEM/F12 (#12634028, Thermo Fischer Scientific, Bremen, Germany), and 20% R-spondin conditioned medium (HA-R-Spondin1-Fc 293T Cells, #AMS.RSPO1-CELLS, AMS Biotechnology, Abington, UK) with 10% bovine serum albumin (BSA), supplemented with N-Acetyl-L-cystein (163.2 µg/mL, #A9165-25G, Sigma-Aldrich, MO, USA), Nicotinamide (1221.2 µg/mL, #N3376-100G, MerckMillipore, Burlington, MA, USA), Recombinant Human Noggin (0.1 µg/mL, #120-10c, PeproTech, Rocky Hill, NJ, USA), TGFβ type 1 activating receptor-like kinase inhibitor (ALK5) A-83-01 (0.211 µg/mL, #SML0788, Sigma-Aldrich), MAPK inhibitor SB202190 (3.31 µg/mL, #S7067, Sigma-Aldrich), Human EGF (0.05 µg/mL, #AF-100-15, PeproTech) and Gastrin (0.02µg/mL, G9145-1MG, Sigma-Aldrich). For colonoid establishment, CGM was supplemented with Glycogen synthase kinase inhibitor CHIRR99021 (1.16 ug/mL, #72052, STEMCELL Technologies, Vancouver, Canada) and Rho kinase (ROCK) inhibitor Thiazovivin (0.78 μg/mL, #72252, STEMCELL Technologies). Colonoids were passaged every 7-8 days at least five times after establishment before carrying out the assays. General experimental outline is shown in **Figure 1A**. For all experiments, colonoids were passaged into single cells and 10,000 cells per 50 μL Matrigel was used. Colonoids were grown in CGM for ten days with selective ROCK-inhibitor Y-27632 (3.203 μg/mL, #1254, Bio-Techne, Minneapolis, MN, USA) supplemented on day one and day three. Differentiation of colonoids was initiated on day ten by lowering Wnt 3A-CM concentration to 5%, withdrawing Nicotinamide and SB202190 factor from the CGM and adding the pan-Notch inhibitor DAPT (4.324 μg/mL, #2634, Bio-Techne) with media change on alternative days. On day 14, A-83-01 was removed from the differentiation media. Tofacitinib Citrate (CP-690550) (25.2 μg/mL, #S5001, Selleckchem.com) or Budesonide (4.3 μg/mL, # S1286, Selleckchem.com) were added to colonoids in differentiation media on days 11, 13, and 14 (**Figure 1A**). Tofacitinib and Budesonide were dissolved in DMSO and 0.033% DMSO was used as vehicle control. TNF (100 ng/mL, #300-01A, PeproTech) and Poly(I:C) (20 μg/mL, #tlrl-pic, *In vivo*Gen) or IFN-γ (10 ng/mL, # 300-02, PeproTech) were added to the differentiation media and 24-hours later the conditioned media and colonoids were collected for downstream assays. Matrigel was dissolved using cell recovery solution (#734-0107, Corning, NY, United States) and colonoids were washed thrice in ice-cold Phosphate buffered saline (PBS) for immunoblotting or PBS with 0.1% BSA for RNA isolation and stored at -80°C. Alternatively, colonoids were prepared for immunostaining as described below.

### RNA Sequencing and Bioinformatics

To extract RNA from colonoids, the RNeasy mini kit (#74106, Qiagen, Hilden, Germany) was used as per the manufacturer’s instructions. RNA sequencing (RNA-seq) of colonoids was performed as described before (43). RNA-seq libraries were generated using SENSE total RNASeq library prep kit with RiboCop rRNA depletion (Lexogen GmbH, Vienna, Austria). Sequencing consisting of 75 single-end reads was performed using Illumina HiSeq4000 instrument (Illumina, Inc., San Diego, CA, USA), and FASTQ files were generated using bcl2fastq 2.18 (Illumina) as per manufacturer’s protocols.

All RNA-seq data analysis was performed in R software. LIMMA linear models with least squares regression and empirical Bayes moderated *t* statistics were used to identify differential gene expression between experimental groups, and Benjamini–Hochberg false discovery rate (FDR) correction-adjusted *P* values ≤ 0.05 were considered statistically significant. Enrichment analyses were generated with MetaCore+MetaDrug™ version 21.3 build 7060. Normalized reads and log2 values for all experiments are mentioned in **Supplementary File SF2**. The RNA-seq dataset for TNF and unstimulated conditions is available under the GEO accession number GSE172404.

### Immunostaining of Biopsies and Colonoids

Patient colonic biopsies were fixed in 10% buffered formalin for 3-6 days as described previously (44). Colonoids collected in 50 μL Richard-Allan Scientific™ HistoGel™ Specimen Processing Gel (#HG-4000-012, Thermo Fisher Scientific, Massachusetts, USA) were fixed for 24-48 hours in 10% buffered formalin as described previously (28, 43). Formalin-fixed paraffin-embedded sections from biopsies or colonoids were treated with Neo-Clear (#1.09843.5000, MerckMillipore) and a series of ethanol for de-paraffinization. For immunohistochemistry (IHC), endogenous peroxidase was quenched with hydrogen peroxide and washed once with deionized water. Antigen retrieval was performed by boiling with citrate buffer (pH 6.0) for 15 minutes in a microwave oven. All samples were blocked with 3% BSA in 0.25% Triton-X in PBS (PBST) for 30 minutes at room temperature, washed twice in PBST, incubated overnight at 4°C with pan MHC-II-Mouse monoclonal Anti-HLA DR + DP + DQ antibody [CR3/43] (1:400 dilution in 1% BSA in PBST, #ab7856, Abcam, MA, USA) in a hydrated chamber. The following day, for secondary staining in immunofluorescence, sections were processed with MaxFluor™ 488 Immunofluorescence Mouse Detection Kit (#MF31-M, Dianova, Hamburg, Germany) as per manufacturer’s protocol and incubated 15 minutes with DAPI (1:1000 dilution in PBS, 1 mg/ml solution, #62248, Thermo Fisher Scientific, Massachusetts, USA). The rabbit/mouse EnVision-HRP/DAB+ kit (#K5007, Dako, CA, USA) was used for IHC, and sections were counterstained with haematoxylin.

### Bright-Field Imaging, Confocal Imaging and MHC-II Quantification

All brightfield images were captured with Nikon E400 microscope, DS-Fil U2 camera, and NIS-Elements BR imaging software (Nikon Corporation, Tokyo, Japan) with 20x objective and processed with Fiji (46). Confocal fluorescence microscopy images were captured with 20x or 63x objective using a Zeiss LSM 880 Fast Airyscan Confocal microscope (Zeiss, Oberkochen, Germany). To minimize photobleaching, laser power typically was 20% under maximum, and the pinhole was set to 0.8–1.5. Images were processed with Zen Blue 2.6 software (Zeiss). Quantitative confocal image analyses of MHC-II staining in colonoids were performed using Fiji (46). The fluorescence intensity of MHC-II staining was determined by measuring the integrated density. Otsu’s thresholding algorithm was applied to convert images with DAPI staining to binary images, and the nuclei (cell counts) were quantified using the Analyze Measure plugin in ImageJ. The corrected total cell fluorescence (CTCF) was calculated: CTCF = Integrated density-(Area of selection X Mean fluorescence of background readings). The average of CTCF/cell counts for five power fields per condition for each experiment is represented in the graphs. A total of 2000-6000 cells in the colonoids were counted per treatment condition. Quantification results are presented in **Supplementary File SF1**, sheet no 3.

### Immunoblotting

Colonoids were taken from -80 °C, washed twice in ice-cold PBS before they were lysed for 2 hours on ice in lysis buffer containing 50 mM Tris-HCl pH 7.5, 150 mM NaCl, 5 mM EDTA, 1% NP-4#0, 1 mM DTT, 1x Complete^®^ EDTA-free protease inhibitor (#11873580001, Sigma-Aldrich), and 1x phosphatase inhibitor cocktail I (#P2850, Sigma-Aldrich) and III (#P0044, Sigma-Aldrich), respectively. Protein lysates were clarified by centrifugation at 14,000 × *g* for 20 minutes at 4°C, and protein concentration was measured using the Bradford protein assay (Bio-Rad, California, USA). Protein lysates were denatured in 1 × NuPage lithium dodecyl sulfate (LDS) sample buffer supplemented with 40 mM DTT for 10 minutes at 70°C before they were separated on 4–12% NuPage Bis-Tris gels (# NP0321BOX, Invitrogen, MA, USA) and electroblotted onto nitrocellulose membranes (Bio-Rad). The membranes were blocked in Rockland Blocking Buffer for fluorescent Western Blotting (#MB-070, Rockland, PA, USA) for 1 hour at room temperature before incubation with the indicated antibodies overnight at 4°C. The blots were incubated with Dylight secondary antibodies (Invitrogen) for visualization. Images were obtained with LI‐COR Odyssey and analyzed using Image Studio Software (LI‐COR Biosciences, NE, USA). Total protein levels were normalized to GAPDH and expressed as fold change to untreated DMSO control. The following antibodies were used: HLA DR + DP + DQ mouse monoclonal antibody [CR3/43] (1:400 dilution, #ab7856, Abcam), HLA Class 1 ABC mouse monoclonal antibody [EMR8-5] (1:200 dilution, #ab70328 Abcam), STAT11 (9H2) mouse monoclonal antibody (1:1000 dilution, #9176, Cell Signaling Technology, Danvers MA, USA), STAT1 Y701:Phospho-Stat1 (Tyr701) (D4A7) rabbit monoclonal antibody (1:1000 dilution, #7649 Cell Signaling Technology), and GAPDH (D16H11) XP rabbit monoclonal antibody (1:5000 dilution, #5174, Cell Signaling Technology). Quantification results are presented in **Supplementary File SF1**, sheet number 4.

### Analysis of IFNγ Protein in Conditioned Medium

IFNγ measurement by ELISA was carried out using IFN gamma Human Uncoated ELISA Kit (#88-7316-86, Thermo Fischer Scientific) as per the manufacturer’s instructions.

### Statistical Analysis

Data from RNA sequencing was analyzed as described above. For all other datasets, statistical analyses were performed in GraphPad Prism 9.0. For normally distributed datasets containing two groups, paired T-test was used, and for normally distributed datasets with more than two groups, one-way ANOVA with Geisser-Greenhouse correction followed by Šidák multiple comparison testing was used. For data not following normal distribution, nonparametric Friedman’s test followed by Dunn´s multiple comparison tests were done. Comparisons between UC and non-IBD controls are made with Two-way ANOVA followed by Tukey’s multiple comparison tests. All datasets with *P* < 0.05 was considered statistically significant and is indicated by *.

## Results

### Differentiated Colonoids Express MHC-II Related Genes at Constitutive Conditions

MHC-II expression by IECs is particularly described in the small intestine, where it is expressed in differentiated cells at constitutive conditions (4) and in a subset of small intestinal undifferentiated epithelial stem cells (12). In colonic epithelium, it is considered to be absent in constitutive conditions and upregulated during inflammation (4, 16, 18). It is unclear if colonoids as a model system express MHC-II genes at constitutive conditions. Therefore, we characterized the expression of MHC-II related genes in differentiated and undifferentiated colonoids using an in-house RNA sequencing (RNA-seq) dataset from 3 non-IBD controls (D1-D3, **Table 1**). Although we noted large interindividual differences in normalized mRNA reads, we found that differentiated human colonoids expressed significantly higher levels of MHC-II related genes *HLA-DMA, HLA-DMB, HLA-DRA, HLA-DRB1*, and *HLA-DRB6* when compared to undifferentiated colonoids. Further, *HLA-DRB5, HLA-DQB1*, and the MHC-II transactivator *CIITA* and MHC-II invariant chain *CD74* had a tendency towards increased expression in differentiated epithelium compared to undifferentiated epithelium (**Figure 1B**). Since MHC-I gene expression is not well characterized in colonoids, we also explored its expression in differentiated vs. undifferentiated conditions. MHC-I gene subtype *HLA-C* was significantly upregulated in the differentiated epithelium, and other MHC-I related genes *HLA-A*, *HLA-B*, non-classical *HLA-E* and *HLA-F*, class I component Beta-2-Microglobulin (*B2M*) showed a similar tendency of increased expression in the differentiated epithelium compared to undifferentiated epithelium (**Figure 1C**). Taken together, differentiated colonoids express higher levels of MHC-II and MHC-I genes than undifferentiated colonoids.

### TNF+Poly(I:C) Stimulation in Colonoids Enhances Expression of Genes Relevant to Antigen Presentation Pathway

**Figure 2:**
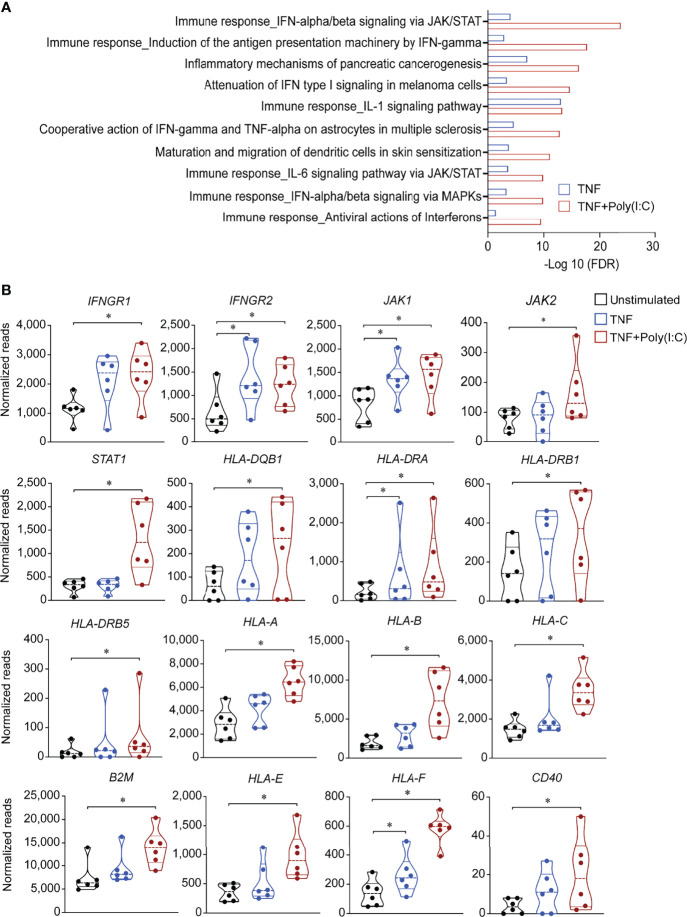
Gene expression alterations in antigen presentation pathways due to TNF+Poly(I:C) stimulation of colonoids. Gene expression data from colonoids (*n* = 6 donors) stimulated with TNF alone (blue) or TNF+Poly(I:C) (red) for 24 hours in comparison with unstimulated (black) colonoids are depicted. **(A)** Enrichment analysis showing top 10 statistically significant (adjusted *P* < 0.05) differentially regulated pathways. Bars indicate –Log10 false discovery rate (FDR). Enrichment analyses are generated with MetaCore+MetaDrug™ version 21.3 build 70600. **(B)** Violin plots depicting normalized reads of genes relevant to antigen presentation pathways regulated by 24-hour stimulation with TNF or TNF+Poly(I:C) compared to unstimulated controls. *P* values are obtained by LIMMA linear models with least squares regression and empirical Bayes moderated t-statistics with Benjamini Hochberg FDR correction for multiple comparisons and * indicates *P* < 0.05.

Next, we looked at another in-house RNA-seq data set from colonoids derived from 3 non-IBD and 3 UC donors (D1-D3 and D7-D9, **Table 1**), stimulated with either TNF alone or in combination with Poly (I:C) (TNF+Poly(I:C)) for 24 hours. Pathway network analysis of the gene expression data revealed that IFN-dependent type I signaling pathway and IFNγ-dependent antigen presentation *via* the JAK/STAT pathways were the most upregulated pathways by the combined stimulation with TNF+Poly(I:C) (**Figure 2A**). Upon further examination of the IFNγ-dependent antigen presentation pathway (**Supplementary Figure 1A**), we observed no detectable levels of *IFNγ* gene expression. IFNγ receptors IFNGR1 and IFNGR2 were upregulated as well as Janus kinase *JAK1*, *JAK2*, and transcription factor *STAT1* were significantly upregulated (**Figure 2B**). Notably, *HLA-DQB1*, *HLA-DRA*, *HLA-DRB1*, *HLA-DR5*, and *CD74*, and MHC-I related genes *HLA-A, HLA-B, HLA-C*, *B2M*, non-classical genes *HLA-E* and *HLA-F*, were upregulated upon stimulation with the combination of TNF+Poly(I:C). Similar tendency for a subset of these genes was observed with TNF-stimulation alone when compared to unstimulated conditions (**Figure 2B**). Costimulatory *CD40* gene expression was also enhanced (**Figure 2B**), and a similar pattern of increased expression was observed for *CIITA* (*P*=0.089) in colonoids stimulated with TNF+Poly(I:C) (**Supplementary Figure 1B**). Since mucosa of UC patients has been shown to express costimulatory CD80 and CD86 that are important for T-cell activation, we looked at their expression in colonoids and found no expression in our RNA-seq data (data not shown). Several other genes relevant to antigen presentation pathway such as *CTSS*, *CD74*, *IRF1*, transcription factor *USF1*, transactivator of MHC-I *NLRC5*, and several proteosome subunits and transporter proteins, were upregulated in the colonoids stimulated with TNF+Poly(I:C) when compared to unstimulated controls (**Supplementary Figure 1C**). Together, TNF+Poly(I:C) stimulation of colonoids leads to enhanced expression of genes relevant to antigen presentation, including MHC-II expression. Interestingly, these effects were in line with upregulation of antigen presentation machinery observed in microdissected epithelium from IBD patients with active inflammation (**Supplementary Figure 1D**) (15).

Since MHC-II expression in intestinal epithelium is known to be induced by the IFNγ from the immune cells (19) and there are no immune cells present in our model, we next asked whether IFNγ could be secreted from the colonoids. We measured IFNγ in conditioned medium from unstimulated and TNF+poly(I:C) stimulated colonoids from *n* = 5 donors (D3, D5, D7, D9 and D10, **Table 1**) by ELISA but could not detect any IFNγ protein (data not shown). This is in line with our findings that *IFNγ* mRNA was not detected in the colonoids.

### TNF+Poly(I:C) Stimulation of Colonoids Induces Apical and Basolateral MHC-II Protein Expression

**Figure 3:**
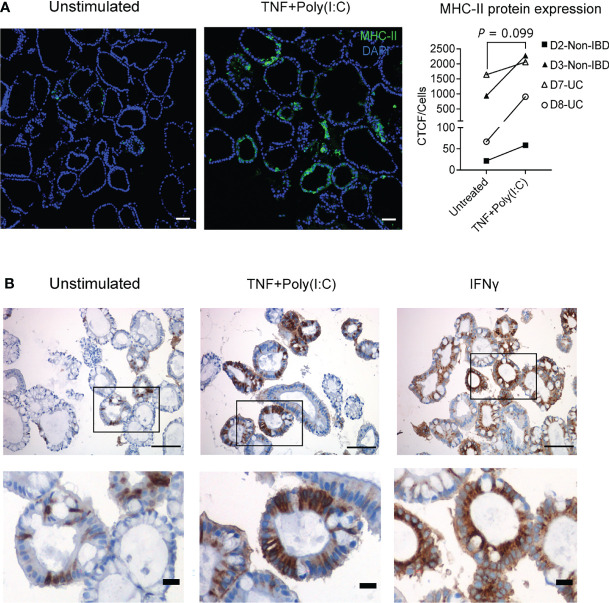
TNF+Poly(I:C)-dependent MHC-II protein expression and localization in colonoids. **(A)** Ligand induced MHC-II protein expression was confirmed by immunofluorescence staining with colonoids from 2 non-IBD (D2-D3) and 2UC (D7-D8) donors. For non-IBD donor D2, this was reproduced in two independent experiments. Representative images are shown on the left. Nuclei are stained blue (DAPI), green colour shows MHC-II protein expression. MHC-II protein quantification as indicated by CTCF/cell counts per condition, for each experiment (averaged counts for D2). Images are obtained with 20x magnification and scale bars indicate 100 µm. *P* values are obtained by paired T-test. **(B)** Immunohistochemistry staining of MHC-II protein localization shown by brown colour in unstimulated colonoids (left), colonoids stimulated with TNF+Poly(I:C) (middle) and IFNγ (right). The colonoids were derived from non-IBD control D2 (representative images from *n* = 3 experiments). Demarcated areas in top row are shown magnified in picture below. Images are obtained with 20x magnification and scale bars indicate 100 µm in the top row and 20 µm in the bottom row.

MHC-II protein expression and localization in IECs is altered during IBD, with enhanced expression observed at basolateral sides of the epithelium that may be functionally relevant for IECs to present antigens to immune cells in lamina propria (47). Therefore, we wanted to evaluate the protein expression and localization of MHC-II expression upon TNF+Poly(I:C) stimulation. Colonoids stimulated with TNF+Poly(I:C) or IFNγ for 24 hours were stained for MHC-II by immunofluorescence (**Figure 3A**) and immunohistochemistry (**Figure 3B**). In accordance with the gene expression data, we found that TNF+Poly(I:C) stimulation had a tendency towards upregulation of MHC-II protein levels when compared to unstimulated colonoids (**Figure 3A**). Immunohistochemistry showed staining of differentiated colonoids from donor D2, confiring that this donor expressed MHC-II protein at untreated conditions (**Figure 3B**, left panels). The extent of MHC-II protein expression was enhanced upon stimulation with TNF+Poly(I:C) and IFNγ (**Figure 3B**, middle and right panels). In line with observation by others (19) the colonoids showed mainly enhanced apical expression of MHC-II after 24 hours of IFNγ stimulation. MHC-II protein appeared to be expressed in both basolateral and apical expression of MHC-II after 24 hours TNF+Poly(I:C)-stimulation (**Figure 3B**, middle panels). Basolateral and apical MHC-II expression was also observed in donors D2, D3, D7, and D8 by high resolution confocal imaging (**Supplementary Figure 2**). Thus, MHC-II protein expression can be induced directly by TNF+Poly(I:C) stimulation at basolateral sides as reported for epithelial MHC-II expression in active IBD (47).

### Tofacitinib Downregulates TNF+Poly(I:C)-Dependent MHC-II Protein Expression

**Figure 4:**
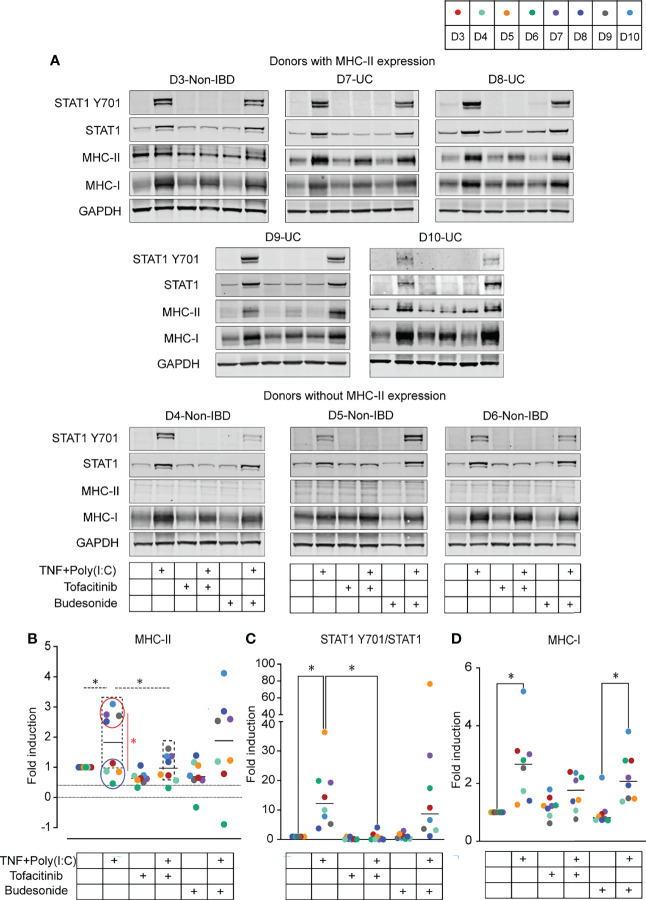
Effect of Tofacitinib and Budesonide pre-treatment on TNF+Poly(I:C)-stimulation in colonoids by immunoblotting. **(A)** Immunoblots from colonoids showing STAT1 Y701, STAT1, MHC-II, MHC-I protein expression in colonoids from donors expressing MHC-II (top) and donors without MHC-II expression (bottom). Immunoblotting quantification of MHC-II protein expression **(B)**, activated STAT1 protein expression represented as STAT1 Y701/STAT1 **(C)**, and MHC-I protein expression **(D)**. Fold expression is generated by normalizing to DMSO unstimulated control for each donor and further normalized to GAPDH expression. In all quantification results, each donor is indicated by colored dots, and donor numbers and colour codes are shown at the top of the figure. Dashed lines represent statistical tests on donors expressing MHC-II at a constitutive level (D3, D7-D10, marked by dashed box). Colonoids from UC donors (D7-D10, red circle) are compared with non-IBD donors (D3-D6, blue circle) and red line represents statistical testing between these groups. *P* values are obtained by one-way ANOVA followed by Šídák’s multiple comparisons test (B-dashed lines), Two-way ANOVA followed by Šídák’s multiple comparisons (B-red line) or nonparametric Friedman’s test followed by Dunn´s multiple comparison tests **(C, D)**. * indicates *P* < 0.05. Data represented from UC donor D7 are averaged from two independent experiments.

Gene expression data from colonoids indicated that upregulation of the MHC-I and MHC-II gene expression was associated with increased *JAK1/2* and *STAT1* expression (**Figure 2B**). Therefore, we investigated whether the pan-JAK inhibitor Tofacitinib could alter MHC-I and MHC-II expression. In comparison, we also investigated the effect of the anti-inflammatory glucocorticoid Budesonide on MHC-I and MHC-II expression. Colonoids from both non-IBD (*n* = 4) and UC (*n* = 4) donors (D3-D6 and D7-D10, **Table 1**) were pre-treated with 50 µM of Tofacitinib or 10 µM of Budesonide on day 11 in differentiation media and refreshed with new differentiation media and drugs on day 13 and one-hour before stimulation with TNF+Poly(I:C) on day 14. On day 15, 24-hours post-stimulation (**Figure 1A**), colonoids were collected and assessed for MHC-II and MHC-I expression by immunoblotting (**Table 1**). Colonoids treated with TNF+Poly(I:C) showed enhanced levels of MHC-II protein (**Figures 4A, B**) in donors with MHC-II expression at constitutive conditions (D3, D7-D10, dashed boxes), verifying gene expression data (**Figure 2B**). In donors lacking MHC-II expression at constitutive conditions (D4-D6), we did not observe enhanced TNF+Poly(I:C)-dependent MHC-II protein expression. There was a significant upregulation of MHC-II protein expression in TNF+Poly(I:C) stimulated colonoids derived from UC donors (red circle) when compared to non-IBD donors (blue circle) (**Figure 4B**). Treatment with TNF+Poly(I:C) in colonoids was also associated with increased activation of STAT1 protein in all donors (**Figure 4C**), in line with the significant upregulation of STAT1 mRNA detected by RNA-seq (**Figure 2B**). Tofacitinib pre-treatment inhibited phosphorylation of STAT1 and reduced the expression of total STAT1 protein levels in colonoids treated with TNF+Poly(I:C) (**Figures 4A, C**). Importantly, Tofacitinib significantly down-regulated expression of TNF+Poly(I:C)-induced MHC-II protein levels in those donors that had MHC-II expression (D3, D7-D10, dashed box) by immunoblotting (**Figures 4A, B**). Budesonide pre-treatment in colonoids neither influenced the TNF+Poly(I:C)-depedent activation of STAT1 protein nor MHC-II protein expression. We also observed TNF+Poly(I:C)-dependent increase in MHC-I protein levels when stained with a pan MHC-I antibody recognizing HLA-A, HLA-B and HLA-C (**Figures 4A**, **Figure 4D**), verifying gene expression data (**Figure 2B**). Some donors showed a tendency towards downregulation of TNF+Poly(I:C)-depdent MHC-I protein levels due to Tofacitinib pre-teatment, but this was not statistically significant (**Figure 4D**). Further, Budesonide pre-treatment did not have any effect on TNF+Poly(I:C)-depdent MHC-I protein levels. Thus, TNF+Poly(I:C)-dependent increase of MHC-II protein expression in colonoids was downregulated with Tofacitinib pre-treatment possibly by repressing activation of STAT1, while pre-treatment with Budesonide had no effect.

**Figure 5:**
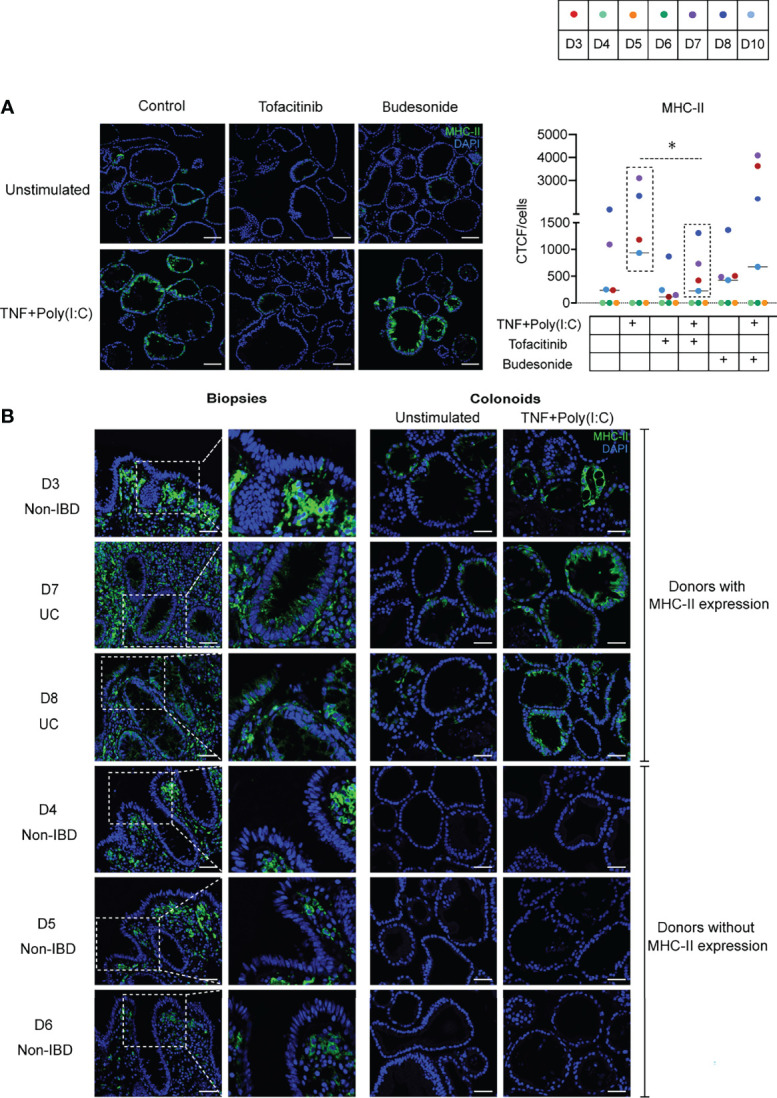
MHC-II localization due to Tofacitinib and Budesonide pre-treatment and comparison with *in vivo* epithelial MHC-II expression. **(A)** Effects of ligand and drug treatment on MHC-II protein expression in colonoids detected by immunofluorescence staining (*n* = 8 experiments derived from 7 donors, for UC donor D8, data represented are averaged from two independent experiments). Representative images are shown on the left. Nuclei are stained blue (DAPI), green colour shows MHC-II protein expression. MHC-II quantification as indicated by CTCF/cell counts per condition for each experiment are shown on the right. Images are obtained with 20x magnification, scale bars indicate 100 µm. In quantification result, each donor is indicated by coloured dots and donor numbers and colour codes are is shown at the top of the figure. As in **Figure 4B**, the dashed lines indicate that TNF+Poly(I:C) induced MHC-II protein expression was significantly downregulated by Tofacitinib in donors that constitutively express MHC-II (D3, D7, D8, and D10, in dashed box). *P* values are calculated by nonparametric Friedman’s test followed by Dunn´s multiple comparison tests and * indicates *P* < 0.05. **(B)** MHC-II protein expression as represented by immunofluorescence staining in *in vivo* biopsies (left panel) and corresponding unstimulated and TNF+Poly(I:C)-stimulated colonoids (right panel) from non-IBD donors (D3-D6) and UC-donors (D7-D8). Areas in the biopsies marked in white dotted boxes are shown adjacent with higher magnification. *In vivo* colonic biopsies for immunostaining from the UC patiens are taken from inflamed area. MHC-II expression is shown in green and DAPI in blue. Scale bar represents 50 µm.

Next, we investigated whether Tofacitinib and Budesonide pre-treatment influence TNF+Poly(I:C)-dependent protein localization of MHC-II. We performed endpoint immunofluorescence staining with the experimental setup described in **Figure 1A**, using a new passage of colonoids from the same donors used for endpoint immunoblotting analysis (**Figure 4**). Tofacitinib pre-treatment downregulated and Budesonide pre-treatment did not affect TNF+Poly(I:C)-dependent MHC-II protein expression (**Figure 5A**) in those donors where MHC-II expression was observed (D3, D7, D8, and D10, dashed box), in line with immunoblotting results (**Figures 4A, B**). We observed apical and basolateral expression of TNF+Poly(I:C)-dependent MHC-II protein expression, as shown in **Figure 3**, but did not find any alterations in protein localization due to the drug treatments.

### Interindividual Differences in *In Vivo* MHC-II Protein Expression Are Mimicked in Colonoids

In our endpoint immunoblotting (**Figure 4**) and immunofluorescense (**Figure 5**) experiments we noticed that MHC-II protein was expressed in colonoids from all UC donors (D7-D10) and one non-IBD donor (D3). Colonoids from the non-IBD donors D4-D6 did not have any MHC-II protein expression (**Figure 4A**). In order to better understand the interindividual differences in MHC-II expression, we examined whether the presence of MHC-II expression in differentiated colonoids mimicked *in vivo* MHC-II expression in colonic epithelium from the same donors (**Table 1**). Amongst the 6 donors compared (4 non-IBD and 2 UC), we observed that colonoids from 1 non-IBD donor (D3) and 2 UC donors (D7 and D8) with MHC-II protein expression in colonoids (**Figure 4**) mimicked MHC-II expression in the colonic epithelium *in vivo* (**Figure 5B**). Further, donors D4-D6 that did not express MHC-II in colonoids also lacked MHC-II expression in the colonic epithelium *in vivo* (**Figure 5B**). Thus, the reproducible interindividual differences in MHC-II expression observed in colonoid experiments recapitulate *in vivo* epithelial MHC-II expression.

## Discussion

In the current study using non-IBD and UC-patient-derived colonoids, we have demonstrated that IBD-associated pro-inflammatory signals TNF and dsRNA (Poly(I:C)) together upregulate MHC-II expression in differentiated colonic epithelium in a pattern similar to that seen in IBD epithelium (15). Importantly, we provide insight into the role of Tofacitinib in downregulating TNF+Poly (I:C) induced MHC-II expression.

Tofacitinib is approved for treatment of ulcerative colitis in patients where first and second line treatments have failed (31, 32). However, the effects of Tofacitinib on IECs remain largely unexplored. The cell permeable small molecule Tofacitinib (CP-690550) Citrate used in our experiments inhibits JAK3 and JAK1 and to a lesser extent JAK2. A decade ago it was shown that CP-690550 reduced TNF-induced synthesis of chemokines like MCP1(CCL2), IP-10 (CXCL10) and RANTES (CCL5) in a selective manner *via* the JAK/STAT signalling pathway since TNF-induced IL-8 (CXCL8) synthesis and secretion remained unchanged (48). Because stimulation of colonoids with TNF+Poly(I:C) was associated with enhanced *JAK1/2* and *STAT1* gene expression, it was of interest to examine the effect of Tofacitinib on the regulation of MHC-II and MHC-I. Tofacitinib has previously been described to regulate processes related to antigen presentation in non-intestinal epithelial cells. It was shown to downregulate IFNγ-induced MHC-I expression in non-small cell lung cancer cell lines (49), and CD80/CD86 in dendritic cells stimulated with lipopolysaccharide, thereby reducing T-cell stimulatory capacity (36). The latter study, however, also showed an unaltered *HLA-DR* expression in DCs upon treatment with Tofacitinib (36). In our dataset, Tofacitinib treatment downregulated TNF+Poly(I:C)-dependent MHC-II protein expression in colonoids. The glucocorticoid Budesonide had no effect on TNF+Poly(I:C)-dependent MHC-II expression, suggesting a significant involvement of JAK1/2-STAT1 signaling pathway in the regulation of MHC-II expression in colonic epithelium. While Tofacitinib has not been directly implicated in the regulation of MHC-II, JAK1/2 inhibitor Baricitinib, a drug approved for Rheumatoid arthritis, has been shown to modulate MHC-II on allogenic antigen-presenting cells and prevent graft vs host disease (50).

Increased MHC-II expression in IECs during active inflammation is considered to be secondary to IFN-γ stimulation from intraepithelial lymphocytes or lamina propria immune cells (19–22). These cell types are not present in our intestinal epithelial organoid model system, and we did not detect IFNγ expression or secretion in our colonoids that could indicate an autocrine loop. We did show that Tofacitinib pre-treatment inhibited phosphorylation of STAT1 and reduced the expression of total STAT1 protein levels in colonoids treated with TNF+Poly(I:C). One possibility is that TNF+Poly(I:C) stimulate the secretion of type I IFNs that can activate the JAK/STAT pathway in an autocrine faschion (28, 48). However, to our knowledge type I IFNs have not been reported to activate MHC-II expression in IECs, and we have not found IFNβ induced protein expression of MHC-II in colonoids (data not shown), as demonstrated for IFNγ. Thus, more studies are needed to define the exact signalling pathways involved in TNF+Poly(I:C) induced MHC-II expression.

Whether or not enhanced antigen presentation in IBD is deleterious or protective is unknown. However, since antigen presentation is a central process in adaptive immunity, the impact of its regulation by Tofacitinib in IBD should be evaluated. Our data indicate that colonoids from donors that expressed MHC-II genes at constitutive conditions showed increased expression in differentiated versus undifferentiated conditions. This is in accordance with data presented by Kelson et al., who also reported *HLA-DR* expression in colonoids derived from pediatric IBD patients (21) and Wosen et al. (19), who showed that differentiated small intestinal organoids express MHC-II in response to IFNγ. Our data did not show a high expression of MHC-II genes in undifferentiated conditions, in contrast to studies observed by Biton M et al. (12), who demonstrated that undifferentiated murine intestinal stem cells express MHC-II. The differentiation protocol used in our study generates organoids containing polarized cells of all the major colonic cell types including absorptive cells, goblet cells and enteroendocrine cells (28). Immunostaining for MHC-II showed that some colonoids expressed MHC-II while some did not. One explanation can be that the there were more fully differentiated cells in the colonoids that expressed most MHC-II, since we showed that differentiated cells express more MHC-II than undifferentiated cells. Interestingly, colonoids from UC donors appeared to have more MHC-II positive cells than colonoids from non-IBD donors, but we need substantially more donors in each group before we can separate between general interindividual differences and differences that are disease specific. Further characterization of MHC-II in specific colonic epithelial subtypes is required for a better understanding of the functionality in colonic epithelium. Studies of interindividual differences in cell composition will also be important to understand the donor heterogeneity observed.

The RNA-seq data demonstrated that differentiated colonoids induce some of the genes in the antigen presentation pathway when stimulated with TNF. Colonoids stimulated with a combination of TNF and Poly(I:C) had upregulation of *IFNGR1* and *IFNGR2*, *JAK1/2* kinases, transcription factor *STAT1* and *NLRC5*, costimulatory genes *CD74* and *CD40*, and several transporter proteins that are relevant to both MHC-II and MHC-I dependent antigen presentation. Importantly, these findings are also captured in microdissected epithelium from IBD patients with active disease (**Supplementary Figure 1D**) (15). Therefore, TNF+Poly(I:C)-dependent expression of antigen presentation pathway may provide a conceptual basis for enhanced antigen presentation involving signaling from DAMPs or viral infections in the context of TNF-driven inflammation during active IBD.

Localization of MHC-II in IECs was shown to be relevant for IBD pathogenesis. MHC-II expression is enhanced at the basolateral sides of the epithelium during active IBD, presenting antigen to the immune cells in lamina propria to facilitate adaptive immunity during inflammation (5, 47). Previous studies show that MHC-II was expressed on the apical side of enteroids stimulated with IFNγ for 24 hours. The authors also showed that long-term stimulation with IFNγ for 72 hours induced basolateral expression of MHC-II (19). Our results however demonstrate that stimulation of colonoids with TNF+Poly(I:C) leads to both apical and basolateral expression of MHC-II already after 24 hours. Apical MHC-II could be important for directly controlling microbiome composition in the colon (38, 51, 52) as well as activation and differentiation of intraepithelial lymphocytes (14). Basolateral MHC-II may be essential for enhancing MHC-II expression on mononuclear phagocytes (38) or presenting to CD4+ T cells in the lamina propria (12, 24). Therefore, apical and basolateral MHC-II may have multiple roles during adaptive immunity in IBD. While the focus of our study is to understand regulation in colonic epithelium, a limitation is that the colonoid model system does not allow for evaluating the functionality of enhanced IEC-specific TNF+Poly(I:C)-dependent MHC-II as well as its regulation by Tofacitinib in immune cells. However, future colonoid-immune co-culture studies may be adopted to address these limitations.

In the present work we performed a series of independent experiments to examine expression of MHC-II with several techniques (RNAseq, Western blot, IHC and Confocal imaging) in colonoids derived from totally ten donors (**Table 1**). We observed that colonoids derived from four UC-patients (D7-D10) expressed MHC-II in all the independent experiments. Thus, we could detect MHC-II expression with different techniques, that was also reproducible in independent experiments using the same detection techniques. Amongst the six non-IBD donors included, two expressed MHC-II (D2 and D3), while four did not (D1, D4-D7). Thus, we conclude that MHC-II expression in intestinal epithelial cells appeared donor-dependent. Importantly, the presence or absence of MHC-II protein in differentiated colonoids could be captured *in vivo* in the colonic epithelium of corresponding donors, showing a major strength of the colonoid model system. In two out of six non-IBD controls and all four UC patients used in this study, we found variable MHC-II expression at constitutive conditions. Donor-specific variation in MHC-II protein expression has also been reported previously (19). Constitutive MHC-II protein expression in colonoids was upregulated with TNF+Poly(I:C) stimulation which was associated with enhanced STAT1 activation. STAT1 activation was also observed in colonoids from those donors that lacked MHC-II expression, after TNF+Poly(I:C) stimulation. Thus, colonoids from all donors responded to TNF+Poly(I:C). MHC-II genes are amongst the most hyper polymorphic genes and are influenced by environmental factors such as microbiome (14, 24, 51) and diet (25). Therefore, the variability in MHC-II protein expression observed in our study could be due to epigenetics, gene variations or other undisclosed factors in the donors. In 2016, Dotti et al. (53) showed that some gene expression differences are maintained in *ex vivo* expanded epithelial organoid cultures generated from biopsy samples of patients with UC compared with non-IBD subjects. The authors concluded that these differences are possibly due to permanent changes in the stem cell compartment and speculated whether epigenetic changes may contribute to expression alterations of the UC epithelium. In future studies it will be interesting to study wether e.g., epigenetic changes triggered in inflamed tissue persist during culture and participate to the interindividual variations in MHC-II expression observed. We also observed donor-dependent variability to Tofacitinib treatment. These results highlight the need for understanding interindividual differences in disease pathobiology and drug responses, which may be achieved by utilizing patient-derived colonoid model systems.

## Data Availability Statement

The datasets presented in this study can be found in online repositories. A subset of the data are available at: https://www.ncbi.nlm.nih.gov/geo/query/acc.cgi?acc=GSE172404.

## Ethics Statement

The studies involving human participants were reviewed and approved by Central Norway Regional Committee for Medical and Health Research Ethics (reference numbers 5.2007.910 and 2013/212/REKmidt). The patients/participants provided their written informed consent to participate in this study.

## Author Contributions

TB supervised the study. SG, MDH, HKS, IAR, AvBG, IB, TB contributed to experimental design, generated, and analyzed data. AEØ and AKS collected and characterized patient samples. SG made figure panels and drafted the manuscript along with TB. All authors reviewed and edited the manuscript. All authors contributed to the article and approved the submitted version.

## Funding

This study was funded by the Faculty of Medicine and Health Sciences, NTNU (SG, HKS, IB, TB, AvBG and AKS), the Liaison Committee between the Central Norway Regional Health Authority and NTNU (SG, MDH, IAR, AvBG, AEØ, IB, AKS, and TB), the Liaison committee between St. Olav’s University Hospital and Faculty of Medicine and Health Sciences at NTNU (TB and AvBG), and the Research Council of Norway (AvBG, FRIPRO 262549). The authors work within the Clinical Academic Group for Precision Medicine in Inflammatory Bowel Disease (CAG-IBD https://www.ntnu.edu/cag-ibd/), which is supported by The Liaison Committee for Education, Research and Innovation in Central Norway (Project no. 90545800). 

## Conflict of Interest

The authors declare that the research was conducted in the absence of any commercial or financial relationships that could be construed as a potential conflict of interest.

## Publisher’s Note

All claims expressed in this article are solely those of the authors and do not necessarily represent those of their affiliated organizations, or those of the publisher, the editors and the reviewers. Any product that may be evaluated in this article, or claim that may be made by its manufacturer, is not guaranteed or endorsed by the publisher.

## Supplementary Material

Supplementary Figure 1 Regulation of antigen presentation genes in colonoids and microdissected epithelium. Gene expression data from colonoids (*n* = 6 donors) stimulated with TNF alone or TNF+Poly(I:C) for 24 hours in comparison with unstimulated colonoids. **(A)** Canonical pathway map generated by MetaCore+MetaDrug™ version 21.3 build 70600 software representing antigen presentation pathway as one of the top pathways regulated by TNF+Poly(I:C) stimulation in colonoids. Bars with red indicate upregulated expression of indicated genes by 1:TNF stimulation or 2:TNF+Poly(I:C) stimulation. **(B)** Violin plots depicting *CIITA* gene expression in colonoids. **(C)** Heatmap depicting log2 ratio of genes relevant to the antigen presentation pathway in TNF or TNF+Poly(I:C)-stimulated colonoids when compared to unstimulated controls. **(D)** Heatmap depicting log2 ratio of genes in microdissected colonic epithelium from active IBD (*n* = 12) compared to uninflamed epithelium (healthy controls and IBD in remission, *n* = 17). Part of this data is also presented in Sæterstad et al. (15),. *P* values are obtained by LIMMA linear models with least squares regression and empirical Bayes moderated t-statistics with Benjamini Hochberg FDR correction for multiple comparisons and * indicates *P* < 0.05.

Supplementary Figure 2 MHC-II localization in colonoids stimulated with TNF+Poly(I:C). Immunofluorescence staining of MHC-II protein in IFNγ or TNF+Poly(I:C) treated colonoids. Nuclei are staining blue (DAPI), green shows MHC-II protein expression in the colonoids which are derived from four donors (D2, D3, D7 and D8, ). Confocal fluorescence microscopy images were captured with 20x and 63x objective. On left images with 20x magnification and scale bar 100 µm. White arrows indicate organoids that are zoomed and presented in the images on the right. Zoomed images on the right are from 20x magnification for IFNγ stimulated colonoids and 63x magnification for TNF+Poly(I:C) stimulated colonoids and scale bars indicate 50 µm.

Supporting Information
**(Dropbox)** Dropbox link: https://www.dropbox.com/sh/a86zh9ocfmlqgi4/AADN3bx8yVwDAL-XV0RPNRX5a?dl=0.
