# Supplementary figures and images for: Tofacitinib Downregulates TNF and Poly(I:C)-Dependent MHC-II Expression in the Colonic Epithelium
Source: Front Immunol. 2022 May 17;13:882277. doi: 10.3389/fimmu.2022.882277 (PMC9152176; doi:10.3389/fimmu.2022.882277)

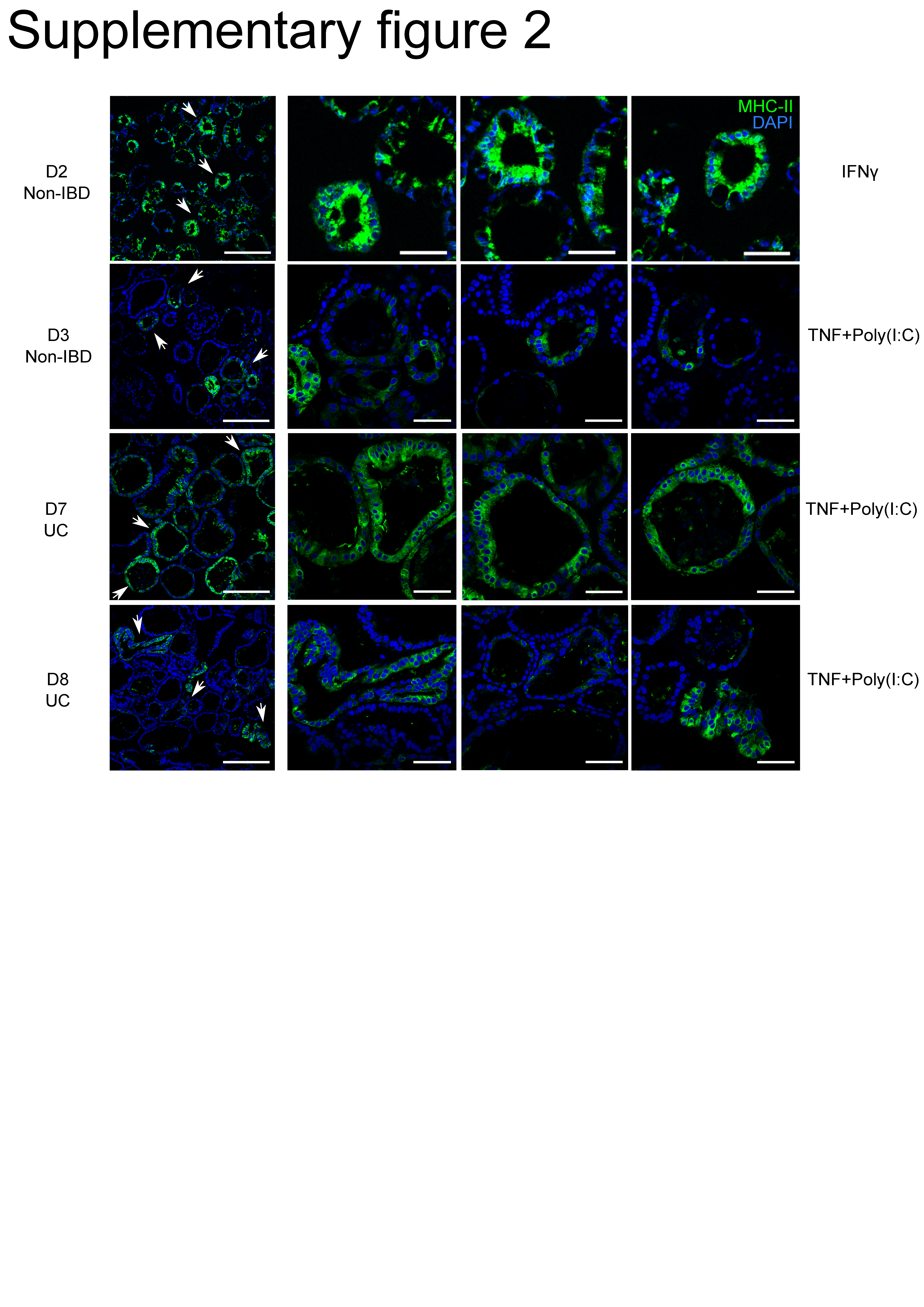

Supplement: Supplementary Figure 2 — MHC-II localization in colonoids stimulated with TNF+Poly(I:C). Immunofluorescence staining of MHC-II protein in IFNγ or TNF+Poly(I:C) treated colonoids. Nuclei are staining blue (DAPI), green shows MHC-II protein expression in the colonoids which are derived from four donors (D2, D3, D7 and D8, ). Confocal fluorescence microscopy images were captured with 20x and 63x objective. On left images with 20x magnification and scale bar 100 µm. White arrows indicate organoids that are zoomed and presented in the images on the right. Zoomed images on the right are from 20x magnification for IFNγ stimulated colonoids and 63x magnification for TNF+Poly(I:C) stimulated colonoids and scale bars indicate 50 µm. [file Image_2.tiff]
